# Investigating local negative feedback of Rac activity by mathematical models and cell-motility simulations

**DOI:** 10.1016/j.isci.2026.114641

**Published:** 2026-01-07

**Authors:** Jupiter Algorta, Jason P. Town, Orion D. Weiner, Leah Edelstein-Keshet

**Affiliations:** 1 Department of Mathematics, University of British Columbia, 1984 Mathematics Road, Vancouver, BC V6T 1Z2, Canada; 2 Department of Biochemistry and Biophysics, University of California, San Francisco, San Francisco, CA 94143, USA; 3 Cardiovascular Research Institute and Department of Biochemistry, University of California, San Francisco, San Francisco, CA 94143, USA

**Keywords:** Cell biology, Mathematical biosciences

## Abstract

How do cells maintain robust, yet flexible polarization for directed motion? Recent optogenetic experiments by Town and Weiner on neutrophil-like HL-60 cells strongly point to the essential role of a Rac-inhibitor (downstream of the small GTPase Rac) in shaping requisite negative feedback that allows cells to respond to rapidly changing directional cues. Here we adapt a previous mathematical model for cell polarity to model interactions of Rac, its putative inhibitor, and upstream PIP3 (a product of the optogenetically stimulated PI3K). We fit parameters in our partial differential equation (PDE) model to temporal and spatial experimental data. Cell shapes, motility, and stimulus responses are modeled in 2D simulations, with PDEs solved along the cell edge. We show that the Rac-inhibitor-PIP3 circuit accounts for the optogenetic data (including exotic cell trajectories), that it is the minimal circuit to do so, and that it improves gradient sensing under noisy or dynamic conditions.

## Highlights

• A mathematical model for Rac GTPase and its inhibitor is simulated along a cell edge
• Model parameters are fit to data for optogenetically stimulated neutrophils
• Simulations of cell motility reveal roles of signaling components in cell responses
• Full model accounts for cell paths in local to global stimulation protocol

## Graphical abstract

**Figure:**
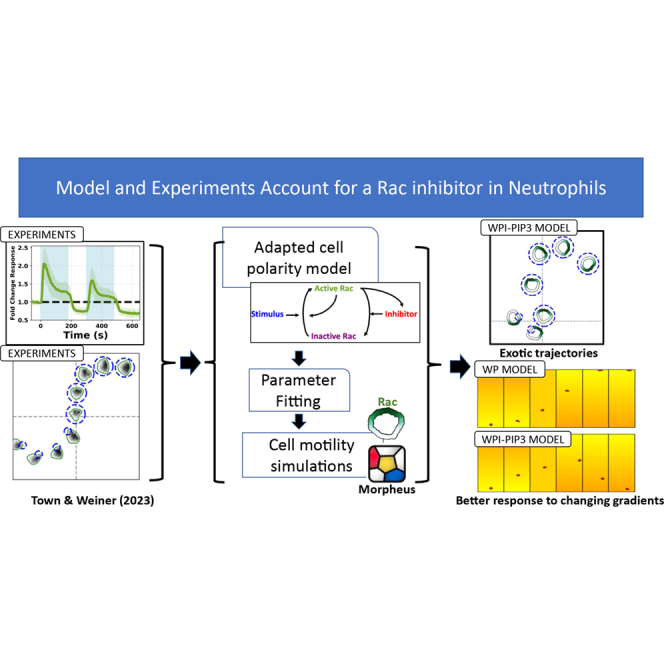

## Introduction

To navigate to sites of injury and infection, neutrophils have to polarize, align with shallow, noisy gradients of chemical signals, and undergo directed migration. It is well known that intracellular components, including the GTPase Rac, play a crucial role in initiating and maintaining cell polarization and transducing external gradients into regulation of the actin cytoskeleton and leading-edge protrusion. The spatial patterning of Rac depends on interacting networks of positive and negative feedback cascades that enable the generation of a dominant front that can be oriented by external guidance cues. *In vivo*, dynamic spatiotemporal gradients of guidance cues and complex 3D environments require the cell to continually adjust its direction of motion, balancing robust polarity with flexible response to its surroundings.

**Figure 1:**
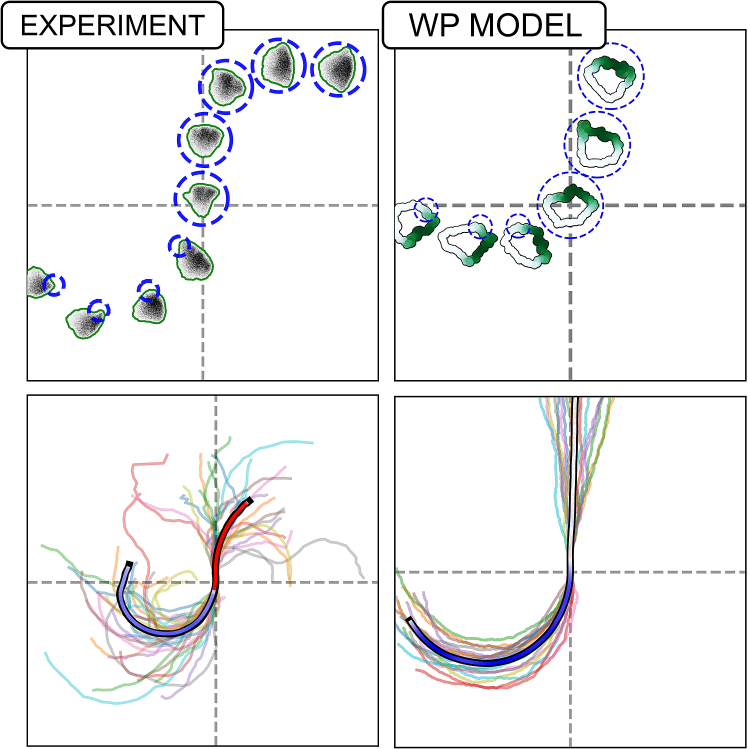
Exotic cell turning is not captured by a basic model Cells stimulated by local followed by global optogenetic stimulation first turn in one direction, then in the opposite direction. Experimental data (left) show the directional behavior and trajectories of cells, with snapshots (top left) and tracked positions (bottom left). The wave-pinning^2^ (WP) model (right) reproduces the initial turning behavior but not the reversed rotation under global stimuli, showing cell shapes and trajectories (top right) and summarized paths (bottom right). In the lower side, all individual cell tracks are shown (25 for the experiment, 20 for the model), with the average trajectory overlaid and color-coded by rotation direction: blue for counterclockwise (CCW), red for clockwise (CW), and white for no rotation. Experimental data adapted from Town and Weiner.^1^ Notably, while experimental cells reverse and begin turning CW after global stimulation, WP model cells continue along a straight trajectory. Video shown in supplemental material: Video S1.

In a recent study, we (Town and Weiner^1^) used computer-controlled spatial optogenetic experiments to test responses of neutrophil-like HL-60 cells to stimuli that mimic reorientation signals *in vivo*. Cells were first polarized and then stimulated locally (on either left or right, or front or rear edges). The light stimulus activates phosphoinositide 3-kinase (PI3K) signaling, known to produce the phosphoinositide PIP3 that, in turn, activates the GTPase Rac, leading to local F-actin assembly and front-edge protrusion. To determine the effect of stimulus history on cell response, cells were first exposed to localized PIP3 stimulus, followed by a global (whole-cell) PIP3 stimulus. This resulted in a zone of Rac inhibition at the previously stimulated site, suggesting a local Rac inhibitor. During motility, cells reverse their direction of turning when the stimulus switches from local to global, suggesting a guidance scheme that measures the local rate of change of the stimulus (see Figure 1, and note that a basic model for polarity fails to explain that observation, as we discuss further on). This surprising result led Town and Weiner to propose a Rac-inhibitor, downstream of active Rac, that builds up alongside Rac zones, to dampen further Rac activation in those locales. While the experiments provide compelling evidence for such an inhibitor, its identity and mode of action remain unknown. Hence, we sought to use mathematical modeling to compare a polarity circuit without the Rac inhibitor (i.e., the wave-pinning [WP] circuit), one with a Rac inhibitor, and another with both the inhibitor and PIP3, where we considered the following questions.

(1) Are there fundamental differences for how cells polarize and move with the Rac inhibitor compared to our previously analyzed polarity circuit^2^?
(2) How does the proposed circuit with the Rac-inhibitor operate in situations where cells experience noisy gradients, or abrupt changes in time-dependent cues? Would the Rac-inhibitor allow for greater flexibility and gradient-following accuracy in the face of noisy, time-varying gradients?
(3) What are molecular properties of the putative inhibitor? Can we infer its rate of diffusion (and hence its molecular weight), its rate of production downstream of Rac, and/or how effectively it inhibits Rac, based on the experimental data?

Video S1. Video comparing WP model vs Experiments in the local-to-global stimulation pattern This highlights how the WP model fails to reproduce the exotic trajcetory seem in experiments.

To address these questions and to rigorously test the proposed hypothesis in Town and Weiner,^1^ we explore a sequence of mathematical models for Rac activity, in which we stepwise add its interactions with the putative inhibitor and with the upstream PIP3. Our original intent was to compare the WP and the WP with inhibitor (WPI) models. As we will show, WPI clearly outperforms WP for much of the experimental data. It later emerged through our simulations that neither WP nor WPI models could account for the more exotic trajectory data, resulting in our third and final model that includes the role of PIP3 (the WPI-PIP3 model). Nevertheless, we have organized the article to demonstrate features that are well-described by the simple models, and to demonstrate where the additional level of complexity was mandated by the data.

Our models represent the time behavior and spatial distribution of signaling components along the edge of a cell before and after optogenetic stimulation. The temporal and spatiotemporal model predictions are compared to the experimental data of Town and Weiner,^1^ and in each case, parameter fitting is obtained by an optimization algorithm. Importantly, we also model the evolving shape and motion of the cell using a well-known method known as the cellular Potts model (CPM). The model reaction-diffusion equations are solved on the cell edge, and regions of high Rac activity are linked to local edge protrusion, while updating the cell shape. In this way, we account for both intracellular Rac signaling and for the cell trajectories and responses to basic and “exotic” protocols.

## Results

### Mathematical models and simulations

To model cell polarization and reorientation, we initially used a simple reaction-diffusion model for active and inactive Rac shown in Figure 3A. This model, originally introduced by Mori et al.,^2^ has the following main assumptions: (1) the total amount of Rac is taken to be constant (on the timescale of interest); (2) inactive Rac diffuses faster than active Rac (serving as a global signal whose depletion limits Rac activation); and (3) active Rac promotes its own activation. In a suitable parameter regime, these ingredients suffice to promote and then stall a wave of Rac activity that resolves into a polar pattern, consistent with the front of a polarized cell.^2^ This partial differential equation (PDE) model is often called the WP model. The simplicity of the WP model and extensive mathematical analysis make it a convenient starting point for our investigation. In the sections that follow, we build on this foundation by introducing additional components to account for more complex behaviors.

### Multiscale simulations in Morpheus

**Figure 2:**
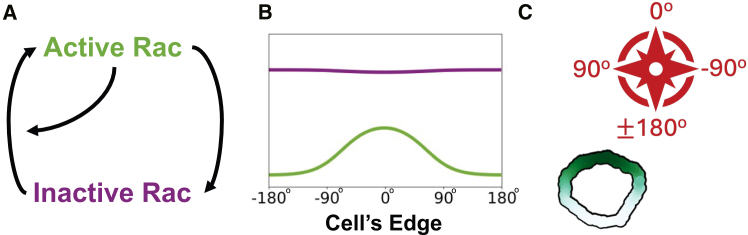
Geometry and basic model setup (A) Basic set of interactions in the simplest model for Rac by Mori et al.^2^ (“wave-pinning”) is formulated as a set of reaction-diffusion equations. (B) The model predicts the distribution of active (green) and inactive (purple) Rac along the cell edge, (a wraparound 1D domain, shown “unwrapped” in B). (C) A polarized cell is represented by the same nonuniform Rac activity distribution along its edge, with high Rac (dark green) defining the cell front (here the cell is polarized northward, i.e., active Rac zone peaks at 0°.) The shape of the cell and its trajectory is simulated in the software Morpheus. The “compass” indicates the lab frame of reference, with 0° corresponding to the northwards direction. In simulations, the cell edge is shown as a wide band for easier visualization of the Rac distribution.

**Figure 3:**
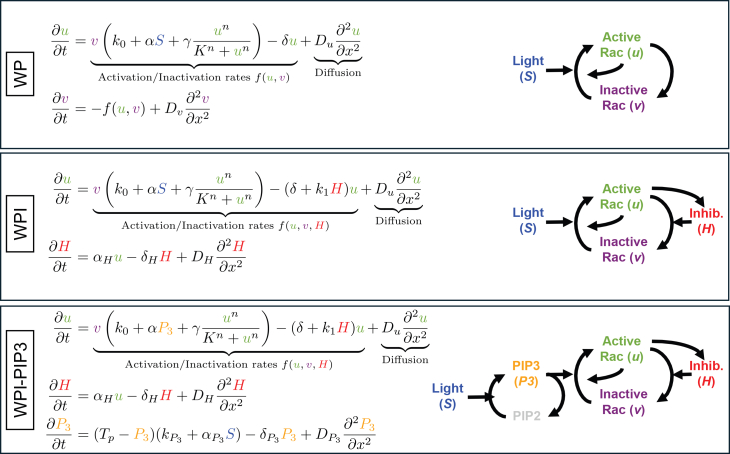
Mathematical models Throughout the study, we focus on three models, (top) WP (wave-pinning), (middle) WPI (wave-pinning with inhibitor), and (bottom) WPI-PIP3 (wave-pinning with inhibitor and PIP3). Parameters described in Table 1. Each model builds upon the previous one with increasing complexity, reflecting the modifications we found necessary to explain experimental results. In the basic WP model (top), we introduced a stimulation term, S, to mimic optogenetic experiments while preserving the original assumption that the reaction kinetics for active and inactive Rac are opposites of each other. This assumption is kept through all models (PDE for inactive Rac is not shown, but included in WPI and WPI-PIP3). In the WPI model (middle), an additional variable (H) represents the local inhibitor of Rac, which is promoted by the active form of Rac. Finally, for the WPI-PIP3 model (bottom), we replaced the direct light stimulation of Rac with light stimulation acting on the PIP3 production, which in turn promotes activation of Rac.

**Figure 4:**
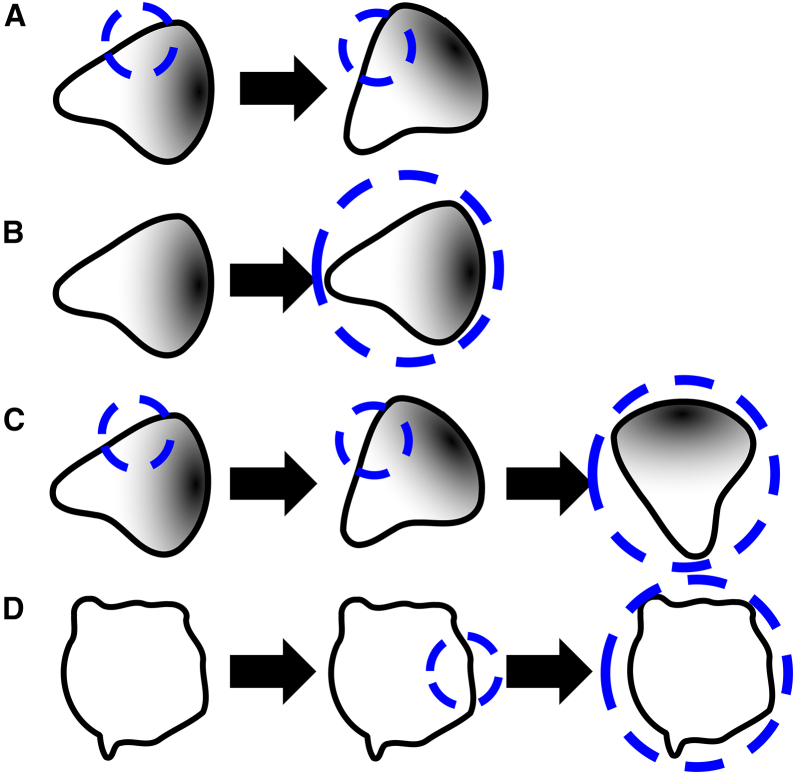
Stimulation protocols The original stimuli in Town and Weiner^1^ included some combination of the above protocols. (A) Local stimulus on the side (or front or rear) of a polarized cell. (B) Global stimulus of a polarized cell. (C) Local followed by a global stimulus of a polarized cell. (D) Treatment of a cell with latrunculin (to disassemble F-actin) followed by local and then global optogenetic stimulus. The location of the stimulus is represented by the dashed circle.

For a multiscale simulation of cell motility that encompasses intracellular signals - we used Morpheus,^3^ an open-source simulation platform based on the CPM. Morpheus allows us to implement the distribution of Rac, its effector(s) and/or upstream signals along a 1D periodic representation of the cell edge in Morpheus, we can also solve the model PDEs on static 2D or 3D cell shapes, but not on deforming domains. Code for the latter is not currently in the public domain. The advantage of the Morpheus platform is that it facilitates the link between the distribution of active Rac and the edge protrusion that dictates the cell’s direction of motion (Figures 2C, 3, 4). The cell’s “front” is defined as the region with the highest concentration of active Rac. Further details on model equations and implementations are provided in the supplemental information.

**Table 1:** Model parameters fit to experimental data

Parameters	WP	WPI
*T* (total Rac) [AU]	9.78 ± 0.70	7.31 ± 0.28
*k*_*0*_ (basal Rac activation rate) [1/s]	0.035 ± 0.006	0.028 ± 0.003
*s* (stimulus-induced Rac activation rate) [1/s]	0.12 ± 0.03	0.059 ± 0.006
γ (autocatalytic Rac activation rate) [1/s]	1.31 ± 0.10	1.38 ± 0.084
*K* (hill-function parameter for autocatalytic) [AU]	2.75 ± 0.19	2.93 ± 0.16
*δ* (basal Rac inactivation rate) [1/s]	2.70 ± 0.11	0.721 ± 0.027
*k*_*1*_ (inhibitor induced Rac inactivation)	N/A	0.372 ± 0.026
*α* (Rac-dependent inhibitor production rate) [1/s]	N/A	6.8 × 10^−3^ ± 4.0 × 10^−4^
*δ*_*h*_ (inhibitor decay rate) [1/s]	N/A	4.0 × 10^−3^ ± 6.0 × 10^−4^

Fitted parameter values for the WP (residuals^2^ = 4,183.57, cells used for fitting = 58) and WPI (residuals^2^ = 615.19, cells used for fitting = 81) models. The stated values and ranges represent a 95% confidence level given the distribution of fitted parameters. The Hill coefficient for Rac autocatalysis was kept fixed at *n* = 2. Time is in seconds (s). Data and models are scaled so that active Rac is normalized (given as fold-multiples of its basal prestimulus level). To capture the heterogeneity among cells, we used the PIP3 data from each individual cell as the input to the WP and WPI models and fitted them to that cell’s corresponding Rac data. This approach was crucial because the PIP3 responses to light stimulation varied strongly between cells, and accounting for these differences was essential to reproduce the distinct Rac activation patterns observed experimentally. The inhibitor is in arbitrary units (AUs). For details about the fitting routine, see the Supplemental Information.

In contrast to other models for cell polarization,^4^^,^^5^^,^^6^^,^^7^ the WP model does not follow the typical Turing activator-inhibitor template, nor the local-excitation-global-inhibition structure.^8^ Rather, it is more closely associated with a “substrate depletion” mechanism, where the reservoir of inactive Rac is used up during Rac activation. Furthermore, WP operates outside the usual “Turing pattern forming” regime, meaning that WP permits both stable resting (uniform) and polarized cell states to coexist, so that cells can respond to signals that are sufficiently large while avoiding a response to small noisy inputs.

The WP model has several parameters that determine its behavior: the basal (*k*_*0*_) and the autocatalytic rates of Rac activation (*γ*), the rate of inactivation (*δ*), and rates of diffusion of the active and inactive forms (*D*_*u*_, *D*_*v*_ respectively). Rac autocatalysis is represented as a saturating (Hill) function of the Rac activity, with a half-max parameter (*K*): the Rac level *R = K* leads to enhanced Rac activation rate of *γ*/2. The values of these parameters were initially set as in Mori et al.^2^ to investigate qualitative model predictions, and then fit to our experimental data, as described below.

### Optogenetic stimulus in the experiments and models

**Figure 5:**
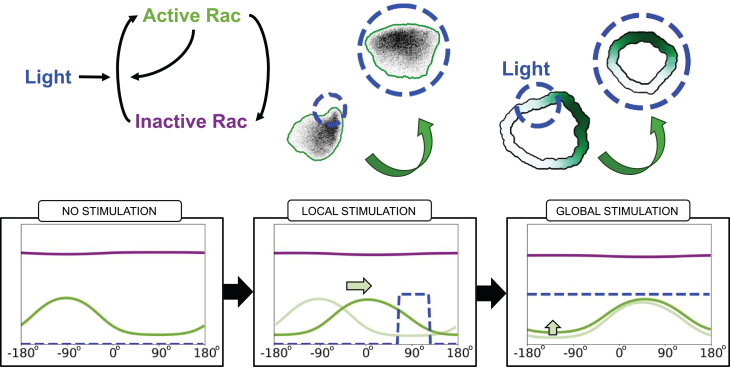
Modeling the optogenetic stimulus The light stimulus is assumed to increase the rate of Rac activation in the models (see Figure 4 and supplemental information). The location of the stimulus is represented by the dashed circle (top). We track the location of the stimulus and the model components in the 1D profile (bottom row) as well as the moving cell-edge simulation. Blue, light stimulus, green, active Rac concentration, purple, inactive Rac concentration along the cell perimeter.

Town and Weiner^1^ express a combination of synthetic proteins in a neutrophil-like cell line. One pair of these proteins enables light-based control over a protein-protein interaction using the iLID system.^9^ By localizing one light-sensitive protein to the plasma membrane and fusing its binding partner to biochemically active “cargo,” the authors were able to recruit the biochemical cargo to the plasma membrane using light, which can be spatially patterned to stimulate some or all of a cell. The biochemically active cargo in this instance was a protein domain known to bind PI3K,^10^^,^^11^ which phosphorylates phosphoinositides and plays an important role in cell polarity. Another set of expressed proteins acted as biosensors, enabling real-time monitoring of stimulated biochemical activity and downstream consequences of that activity. By combining image processing and automated light patterning, Town and Weiner were able to create reproducible optogenetic stimulation protocols. To model the effects of the optogenetic stimulus, we incorporated an additional rate of Rac activation at the site on the cell edge affected by the stimulus. Figure 5 illustrates two types of stimuli used in this study.

(1) Local stimulus that activates Rac in a specific region of the cell edge.
(2) Global stimulus that activates Rac across the entire cell edge.

The profiles of active and inactive Rac are shown (green and purple, respectively) in the images at the bottom of Figure 5, under three conditions: no stimulus, localized stimulus, and global stimulus. The light stimulus is shown in blue.

### The elementary polarity model (WP) accounts for simple turning behavior

**Figure 6:**
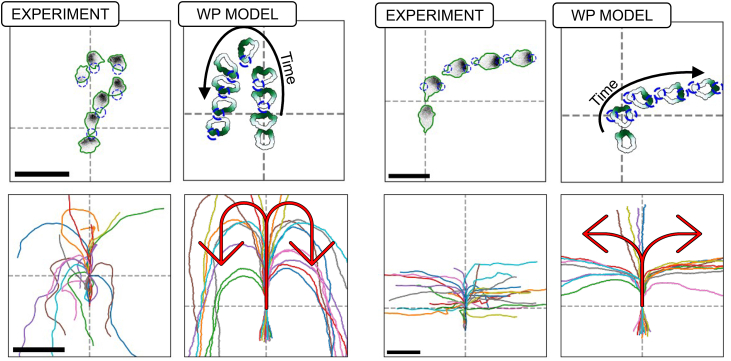
The WP model predicts cell responses to simple stimuli The simplest model for Rac (WP) can satisfactorily account for cell motility responses to basic local optogenetic stimuli. Left two columns, experimental data and model predictions for local rear stimulation; the cells make U-turns and move in the reverse direction. Right two columns, competing two-sided local stimuli; the cell selects one or the other side and turns. Scale bars: 50 μm. This demonstrates that, qualitatively, the WP model can reproduce the observed experimental cell behavior for basic stimuli. The dashed axis indicates where the stimulation started. Video shown in supplemental material: Video S2.

We first tested the simple WP model of Mori et al.^2^ and its predictions for the polarity and motion of a cell exposed to basic local optogenetic protocols shown in Figure 6. As shown in Mori et al.^2^ and subsequent modeling work,^12^ a zone of active GTPase can be shifted by a stimulus that affects the GTPase activation rate. Recall that in experiments (labeled “experiment” in Figure 6) the local stimulus at the rear of a polarized cell will cause the cell to reorient and make a U-turn, while two local stimuli at opposite sides of the cell will result in one or another direction (right or left) “winning”, and cause the cell to turn right or left. We implemented the WP model along the edge of a model cell in Morpheus (as described in methods and in the SI). We found that the WP model accounts for such behavior, in agreement with experimental results, as shown in Figure 6.

Video S2. WP model sucessfully captures the behaviour shown in experiments for: Back stimulation (top) and side stimulation (bottom)

### The Rac inhibitor is required to fit time-dependent data

**Figure 7:**
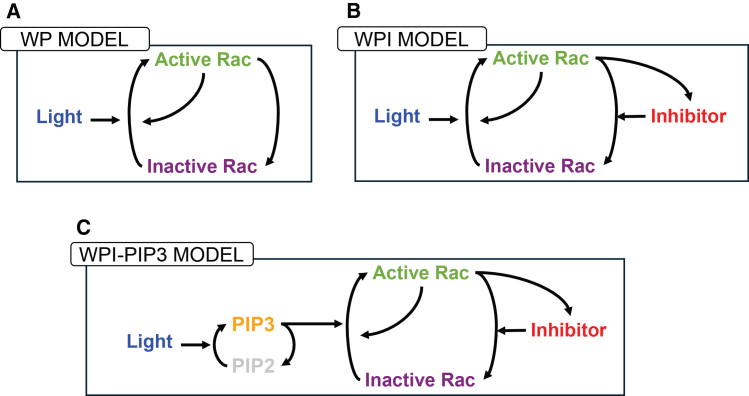
Schematic diagrams for the three competing models (A) The simplest model for Rac activation follows the “wave-pinning” (WP) polarity model of Mori et al.^2^ (B) A Rac-associated inhibitor, proposed by Town and Weiner^1^ is included in the WPI (Wave-Pinning with Inhibitor) model. (C) The role of PIP3 was also considered in the WPI-PIP3 model. All models can account for simple response to single stimuli, but only the WPI-PIP3 model can also account for the behavior observed in the local to global stimulus experiment.

**Figure 8:**
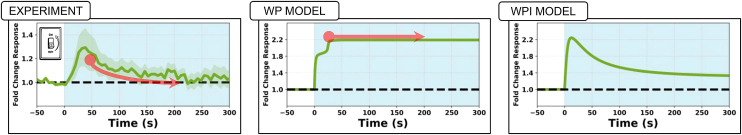
Experiment and model responses to stimulus turned ON Active Rac fold change over time averaged over the whole cell, shown in green. Left, experimental data for an “ON” switch pulse stimulation of latrunculin-treated cells. Center, WP model predictions. Right, WPI model predictions. WPI is qualitatively better, accounting for the decay following peak Rac activity seen in the data (as highlighted by the red curves). PIP3 levels quantified in the experiments were used as a direct input to Rac (as surrogate for the light stimulus) for the WP and WPI model.

To test the idea of a Rac inhibitor, we revised the model, as shown in Figure 7, B. We added a PDE for the inhibitor (*H*), assuming that it is produced at a rate proportional to the level of active Rac, and that it decays with first-order kinetics. We also assumed that the inhibitor diffuses with some rate *D*_*H*_. We used the time-dependent on-off pulse experiment to assess the ability of the WP and WPI models to qualitatively explain the data. Briefly, cells were exposed to a light stimulus that was turned “ON” instantaneously. The data displays features of adaptation,^13^ including a rise in Rac activity, followed by decay back to its basal level. Experimental observations (left), as well as predictions of the WP (center) and WPI (right) models are shown in Figure 8, where the red curves highlight this qualitative feature. We observe that the WPI is a better fit to the time dynamics than the WP model. In particular, the WPI model can account for the overshoot of Rac, whereas WP cannot.

### Fitting model parameters to the data for time dynamics in latrunculin-treated cells

Town and Weiner^1^ recorded cell trajectories, stimulation patterns, and biosensor responses, and these were used in this study to fit parameters to our models.

Before investigating the full responses of motile cells, Town and Weiner simplified the experimental system to showcase the temporal changes that take place in response to stimuli. To do so, they treated cells with latrunculin (Lat) to immobilize them and abrogate the effect of filamentous actin. Hence, in these experiments, the cell does not deform, and membrane tension plays no role in the dynamics of Rac. The time-dependent levels of PIP3 and active Rac were quantified experimentally by Town and Weiner.^1^ Their data provide an opportunity to fit the models’ temporal behavior before trying to understand the full spatiotemporal dynamics.

To fit parameters to the time-dependent models, we dropped the spatial (diffusion) terms, retaining ordinary differential equations for Rac (active and inactive forms), and its inhibitor. We used a parameter fitting routine, differential evolution,^14^ to find best-fit parameters for each of the two models (WP with 58 cells used for fitting and WPI with 81 cells used for fitting). Fewer cells could be successfully fit to the WP model, due to the sensitivity of WP to parameters. To compare to the experimental data, the model variables were scaled so that the unstimulated uniform active Rac level was set to “1.0” (baseline level) and its stimulated level treated as a “fold multiple” of that basal level.

We used a second improvement for fitting the data: the PIP3 data (rather than the optogenetic on-off timing) was used as a direct input to the models’ Rac activation rate. We also considered the heterogeneity of cells displayed in the data. Details of the fitting method are given in the SI, and an interpretation of the parameter values is provided in a later section.

**Figure 9:**
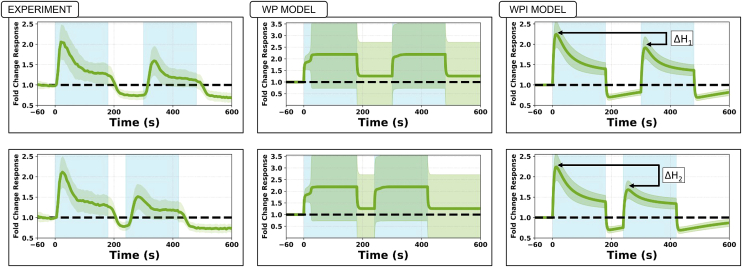
Double-pulse experiment Data^1^ (left) and model fits by the WP model (center, 58 cells used for fitting) and the WPI model (right, 81 cells used for fitting). Mean active Rac (solid green curves) ±1 SD (green shading). Light blue represents stimulus ON phase. Top, a longer delay (Δt_1_ = 120 s). Both models were independently fit to the data from the longer delay (Δt_1_ = 120 s) experiment. Bottom, A short delay, (Δt_2_ = 60 s) between stimuli. The results for the shorter delay (Δt_2_ = 60 s) were generated as predictions, using the fitted parameters. The longer delay leads to smaller difference in amplitudes (ΔH_1_ < ΔH_2_). The WPI, but not the WP model accounts for these experimental features. The WP model fails to capture the heterogeneity of the data, resulting in a wider spread. This also reduced the number of successful fits from a total of 81 (successful WPI model fits) to only 58 (for WP).

The stimulus used to fit parameters to data for Lat-treated cells was a double-pulse, consisting of two ON-OFF step functions, either *Δt*_*1*_ = 120 s or *Δt*_*2*_ = 60 s apart (top and bottom rows, respectively in Figure 9). We compare the experimental data (left) in Figure 9 to model fits. It is typically observed that the second peak of Rac activation is smaller than the first peak, and significantly smaller if the pulses occur in rapid succession.

The parameters found from the fitting procedure were used to solve the WP and WPI models and resulted in predictions shown in the center and right columns of Figure 9. The WPI (but not the WP) model was able to account for several features of the experimental observations, such as the relative height of the first and second peaks (*ΔH*_1_, *ΔH*_*2*_, see right), and the “undershoot” of Rac activity (which fell below its unstimulated level) between and after the stimuli. The WP model failed to reproduce these observations.

Table 1 summarizes the best-fit values of model parameters obtained by the fitting algorithm. We used only the double-pulse experiment shown in the lower left side of Figure 9 (for *Δt*_*1*_ = 120 s) to fit parameters and then simulated the other cases with the set of parameters so obtained. Details about the method are given in the supplemental information.

### Selection of best fit time-dependent model

The time-dependent WPI model has three parameters more than the WP model, as seen in Table 1. We asked whether the WPI model is a better parsimonious fit to the data despite this increase in degrees of freedom. To address this question, we used the Akaike information criterion (AIC)^15^ which rewards a low sum of squared errors and penalizes the number of parameters in comparing distinct models fit to the same dataset. The details of how we computed the AIC score are given in the supplemental information. Lower AIC indicates a better trade-off between fit and complexity and residual sum of square is the residual sum of squares, smaller is better.

### The WPI model responds to stimulation rate

**Figure 10:**
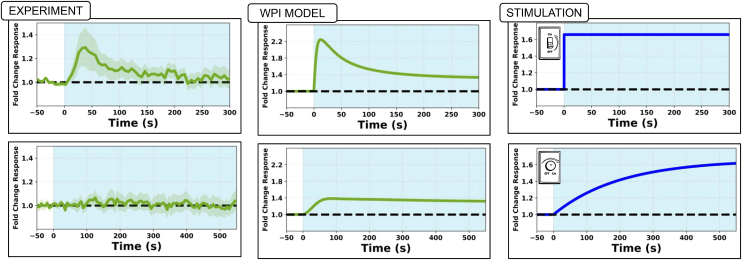
Responses to ON versus ramp stimuli The WPI model distinguishes between abrupt and gradual stimuli (center). A step-function stimulus (top) triggers an overshoot in Rac activity, followed by adaptation, qualitatively agreeing with experiments^1^ (left). A gradual stimulus (bottom) leads to a mild Rac increase above baseline (in contrast with the baseline data) with no overshoot. The findings suggest that the Rac inhibitor filters stimuli, reacting to rapid, rather than gradual changes.

Based on another set of experiments from Town and Weiner,^1^ we investigated the WPI model’s response to the rate of stimulation. Building on results of Figure 8, we asked how the WPI model responds to a gradual “ramp-up” stimulus, in place of the previous ON-OFF pulses. As shown in Figure 10 (bottom row) the WPI model predicts a controlled rise in Rac activity without the overshoot seen in the stimulus pulses (Figure 10, top row). However, the model also predicts a slight increase in Rac activity above pre-stimulus levels, contrary to experimental observations, where Rac levels remain close to baseline.

These findings suggest that the WPI model responds more strongly to rapid stimuli than to gradual changes. Thus, it appears that the inhibitory component serves as a filter, enabling the cell to differentiate effectively between rapid and gradual changes in its environment.

We next sought to determine how the temporal response of the WPI model to stimulation rates translates into a spatiotemporal response to localized stimuli along a cell’s edge. Consequently, we simulated the spatial (PDE) version of the WPI model and examined its response to spatially localized stimuli that were either instantaneous (pulse) or gradual (ramp) in time.

**Figure 11:**
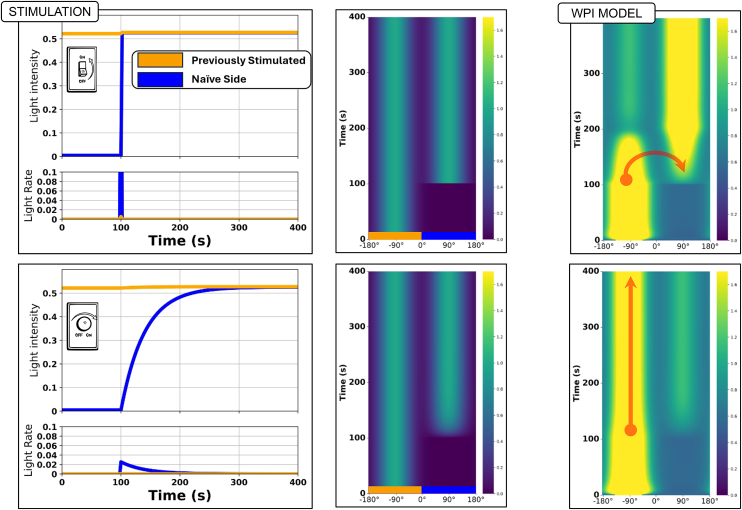
Spatial responses to abrupt versus gradual stimuli The WPI model differentiates between abrupt and gradual stimuli in a spatial context. Model cells were initially exposed to a constant stimulus on one side, followed by the application of a competing stimulus on the opposite side. The competing stimulus had a Gaussian profile, so a small portion bled into the previously stimulated side, producing a very minor upward kink in the traces on that side (left). When the competing stimulus was introduced as a step-function (top), it redistributed the active Rac to its location. Conversely, when the competing stimulus was applied gradually (bottom), the location of the active zone of Rac remained unchanged.

As shown in Figure 11, we initiated the model with a constant stimulus at −90° on the 1D wrap-around cell edge domain. At *t* = 100 s, the second stimulus, introduced at 90° was either a pulse (top) or a gradually increasing stimulus (bottom), implying high and low rates of change, respectively. Despite both sites eventually receiving equal levels of stimulation, the responses (shown as kymographs in Figure 11) were distinct.

In response to the abrupt second stimulus (Figure 11, top row), Rac activity rapidly shifted from its previous site (−90°) to the newly stimulated site. In contrast, in response to the gradual input (Figure 11, bottom row), the original site retained its Rac activity, abrogating the formation of the competing new Rac zone at 90°. These results reinforce the idea that the WPI model responds more strongly at sites on the cell that experience more rapid stimuli, over those regions that experience sustained, gradual stimuli, i.e., that the rate of change of input is the key signal.

In contrast to the WP model where the intensity of the stimulus and/or its time duration is the signal to repolarize,^16^ in the WPI model, the Rac inhibitor effectively filters out gradual stimuli, preserving existing zones of Rac activity unless a rapid perturbation occurs. This mechanism could be biologically relevant in guiding directional responses, allowing cells to ignore weak or slowly changing signals while rapidly adjusting to new, more urgent cues.

### When the stimulus ends, WPI cells keep rotating

We asked whether the WPI model can account for the simplest turning responses. As before, we simulated the CPM cell in Morpheus, this time with the WPI model regulating protrusion along the cell edge. We found that WPI (not shown) can account for simple turning just as well as the WP model shown previously in Figure 6.

**Figure 12:**
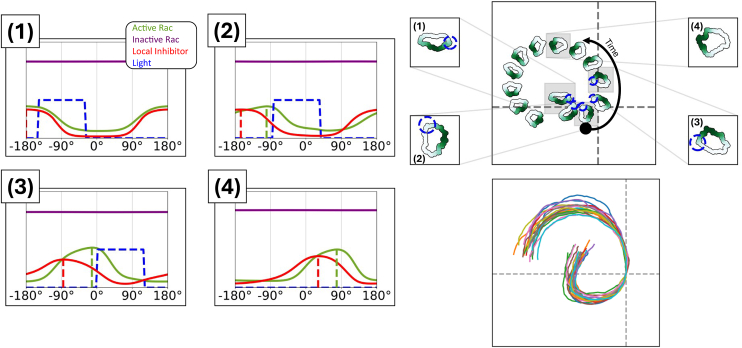
The WPI cells keep rotating after the stimulus is turned off The WPI model is shown, implemented on the “unwrapped” 1D cell edge (left four images) and on a CPM simulation in Morpheus (right). (Left) four time points showing spatial profiles of active and inactive Rac (green, purple, respectively), the Rac inhibitor (red), and the light stimulus (blue). The peaks of active Rac and inhibitor are indicated (green and red dashed lines). (Top right) snapshots of the motile cell moving in 2D at corresponding time points. Active Rac is shown in green shading, and blue circles indicate light stimulus. The inhibitor trails behind Rac, sustaining a persistent rotation of the cell. Time points denote (1) period of initial stimulation, (2, 3) periods during stimulation, (4) period after stimulation. Video shown in supplemental material: Video S3.

In response to a more complex stimulus designed to guide the cell into rotation, we observe a more intriguing result. Initially, we stimulate the cell on its left side (90^°^ counterclockwise from its movement direction), causing the cell to rotate (shown in Figure 12). When the cell’s front was facing north, we stopped the stimulation. As previously shown in Figure 9, Rac activation responds faster to the stimulation than the inhibitor. Spatially, this means that the inhibitor lags behind the Rac zone, effectively pushing Rac forward and sustaining its motion. Correspondingly, we found that the WPI model predicts that cells continue to rotate even after the optogenetic stimulus is turned off. This prediction is in contrast with the experiments, where cells lose polarity and stop migrating after the stimulus is turned off. We can summarize this result by noting that the WPI model can sustain both static polar patterns and traveling waves (TWs) of Rac activity that move around the cell edge (leading to cell rotation). While it is beyond our scope here, we showed in Hughes et al.^17^ that models with very similar negative feedback structure have realms of coexistence of such TW and polar patterns. The stimulus strength required to switch between one and another of these behaviors depends on the strength of the negative feedback and other model parameters.

Video S3. Video showing novelty result of the WPI model in the local-to-none stimulation pattern Cell persists in rotation after the stimulation is removed.

### Fitting spatial Rac polarization data to motile cells

So far, we have only fit parameters to temporal data and non-spatial models for Rac activity in immobilized latrunculin-treated cells. Next, we examine spatial models using data from motile cell polarization which, for the cells we study here, depends on F-actin activity to maintain distinct front and back regions. This aligns with the WP model, where polarization results from different diffusion rates of active and inactive Rac. Inactive Rac diffuses quickly, staying nearly uniform across the cell, while active Rac forms a polar pattern. As polarization occurs, inactive Rac is depleted from the cytoplasm, preventing new polarization sites. Prior studies^2^^,^^17^ noted that the WP model is rather sensitive to parameters. In our hands, the WP model indeed proved difficult to fit reliably to spatial data. We, therefore, proceeded with the WPI and WPI-PIP3 models, keeping all parameter values that we fit from the time-dependent model.

Fitting the spatial data requires the full spatiotemporal model to account for the spatial spread of both Rac activity and Rac inhibitor. Hence, rates of diffusion for these variables also have to be estimated or fit. We report relative diffusion ratios normalized to the active Rac diffusion rate (set to 1), physical units and scaling details are provided in the SI. Fitting was performed across multiple spatial stimulation protocols. Further details can be found in the supplemental information. Similar to the previous fitting, our results aligned with theoretical expectations^2^^,^^17^: (1) the diffusion rate of active Rac is found to be small, while inactive Rac has a much larger diffusion coefficient (approximately 6.40-fold higher in the WPI-PIP3 model and 3.37-fold higher in the WPI model); and (2) the hypothesized inhibitor has a diffusion rate lower to that of active Rac, reinforcing its local nature (Table 2).

**Table 2:** Relative rates of diffusion fit to experimental data

Parameters	WPI	WPI-PIP3
*D*_*u*_ (active Rac diffusion rate)	1	1
*D*_*v*_ (inactive Rac diffusion rate)	3.37	6.40
*D*_*H*_ (inhibitor diffusion rate)	0.14	0.47

Setting the diffusion coefficient of active Rac to D_u_ = 1 (in arbitrary units), we scaled all other diffusion coefficients relative to D_u_. Fitting was performed across all spatial stimulation protocols (166 cells used for fitting), using the average Rac profile within each protocol. As expected, inactive Rac diffuses faster than active Rac. We also found that the inhibitor diffuses most slowly. See SI for correspondence to unit-carrying values and for further detail.

Rates of diffusion (in μm^2^s^−1^) that were fit for the various previous model components differ vastly. For the Otsuji model, the ratio of rates of diffusion of V and U was taken to be 106, much higher than needed by the WP model. For the Meinhardt model, the ratio D_A_/D_C_, was on the order of 0.5. Since these differ greatly from ours and from one another, it is not possible to draw meaningful comparisons for such parameter values.

### WPI does not account for local to global stimulus experiment

Recall that the basic WP model was unable to reproduce the reversal of cell rotation observed experimentally when stimulation switched from local to global (Town and Weiner^1^; Figure 1), implying that additional regulation downstream of Rac was required. Based on arguments in Town and Weiner,^1^ we initially hypothesized that the WPI model would suffice to capture this behavior based on two key findings: (1) Figure 10 shows that the WPI model responds to stimulation rates, suggesting that abrupt stimuli should lead to significant changes in Rac activity; and (2) Figure 11 demonstrates that rapid spatially localized stimuli would allow a new Rac zone to gain advantage and grow, despite continued stimulation of a pre-existing zone.

Given these results, it stands to reason that an abrupt transition from local to global stimulus should leave the original (left) Rac zone unchanged, while promoting a new Rac zone in the (uninhibited) right region of the cell. We argued that, in principle, this sudden change would cause the new Rac zone to outcompete the existing zone, relocating the “front” of the cell, and driving the reversal of cell rotation seen in the experimental data.

To test this, we implemented the local-to-global stimulation protocol as in experiments. To ensure a continuous turning, we stimulated the cell at a site 90° counterclockwise from its front (previously denoted as the “left side of the cell”). After a set time, the local stimulus was abruptly switched to a global one. This rapid switch should have introduced a large, sudden rate of Rac activation at 90° clockwise of the existing front, potentially triggering the cells’ rotation to be reversed. To reflect biological variability across cells, we sample from the pool of fitted parameter sets when simulating multiple trajectories, allowing us to explore population-level responses and heterogeneity in behaviors such as polarization or reversed rotation.

**Figure 13:**
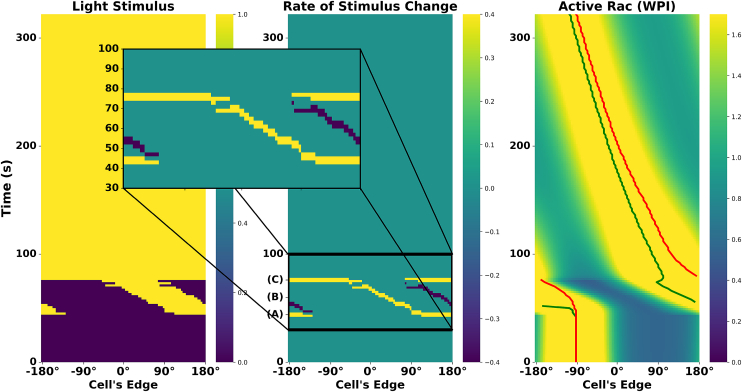
WPI model response to the local-to-global experiment Left to right: kymographs of light stimulus, its rate of change, and active Rac. The zoom-in highlights three key moments: (A) onset of local stimulation, (B) transient phase during rotation, and (C) switch to global stimulation. The WPI model fails to reverse Rac polarity following global stimulus, despite a sharp rate of change at the back of the active zone. Green and red curves in the right side track the peak positions of Rac and the inhibitor, respectively.

Surprisingly, the WPI model failed to reproduce the local to global stimulus experiment. Instead, it persists in counterclockwise rotation, in contrast to the experimentally observed switch. Figure 13 illustrates this result, showing the light stimulus, its rate of change, and the resulting active Rac around the cell’s edge. Peaks of active Rac and inhibitor (indicated with green and red lines, respectively) show how the inhibitor lags behind Rac.

Key insights from these results suggest that while the rate of change in stimulus plays a crucial role (as seen in Figure 11), it is not the only factor governing Rac redistribution. From Figure 12, we learned that Rac maintains persistent rotation due to the timescale differences between its activation and inhibitor dynamics (Table 1). This insight confirms that the inhibitor lags behind Rac with a strong enough influence to prevent the reversal of the cells’ rotation.

Although the rate of change at the right of the Rac zone is indeed large and rapid, it occurs over a very short period of time. The inhibitor, on the other hand, exerts an effect over a longer period, overcoming the transient stimulus shift and preserving the original rotation.

These findings reveal a fundamental limitation of the WPI model: while it captures stimulus-dependent Rac redistribution, it fails to account for persistent inhibitory lag that appears to be crucial for explaining the local to global stimulus experiment.

### Lag due to PIP3 explains local to global stimulus experiment

Having established that the WPI model alone fails to capture the experimentally observed reversal (Figure 13), we sought a minimal extension that could explain this behavior. Our previous results (Figures 9 and 10) demonstrated that Rac redistribution is sensitive to the rate of stimulus change, but in Figure 13, we saw that this effect alone was not sufficient to drive reversal of the cells’ rotation. Instead, Rac persisted in its original rotation.

The data in Town and Weiner^1^ includes the time course of PIP3 upstream of Rac activation. Including this component in our model brings us to the WPI-PIP3 model, which successfully produces the local to global stimulus experiment track. Having a parametrized set of models allows us to ask what features, and under what conditions, would the expanded WPI-PIP3 model account for the reversed rotation. During local stimulation, PIP3 accumulates, thus forming a localized peak. When the stimulus switches to global illumination, this peak remains temporarily stationary and does not dissipate immediately. The lingering PIP3 peak continues to activate Rac locally, while the inhibitor briefly sustains rotation in the same direction (as shown in Figure 12). As Rac progresses, it leaves the PIP3 peak behind, which transiently “pulls” Rac back toward it. This creates a tug of competing influences: the delayed inhibitor pushing Rac forward and the PIP3 peak pulling it backward. As the inhibitor catches up, the PIP3 influence dominates, leading to reversed rotation.

**Figure 14:**
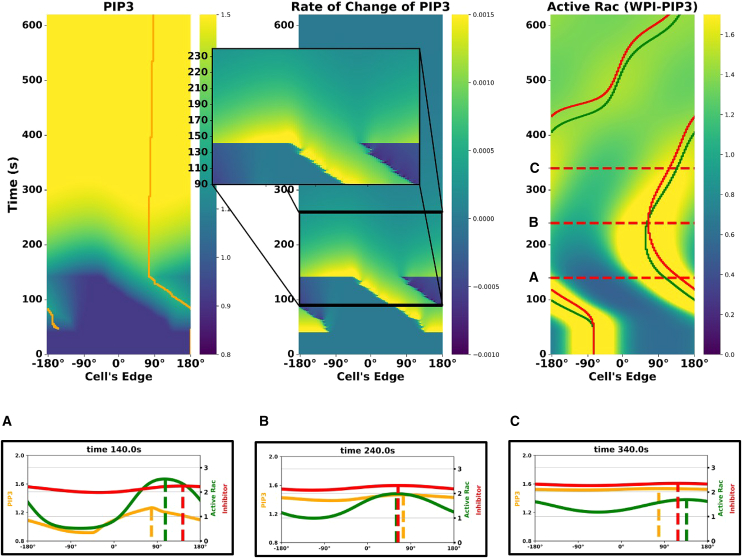
The WPI-PIP3 model accounts for the local to global stimulus experiment (Top) Kymographs of: PIP3 concentration (left), PIP3 rate of change (center), and active Rac (right), with orange, green, and red curves tracking the peaks of PIP3, Rac, and inhibitor, respectively. Time points (on kymograph, right and bottom row): (A) global stimulus turned on, (B) Rac and PIP3 peaks switch positions, (just before the inhibitor catches up) slowing down the movement of active Rac, and (C) reverse rotation initiated. The spatial profiles of PIP3 (orange), active Rac (green), and inhibitor (red) are shown at corresponding time points (bottom row). Dashed lines denote peaks of PIP3 active Rac and inhibitor.

**Figure 15:**
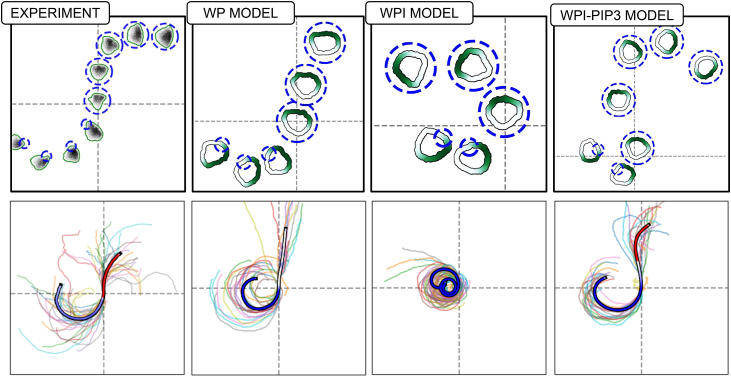
The WPI-PIP3 (but not WP or WPI) model accounts for local to global stimulus experiment A comparison of cell shapes, active Rac distributions along the cell edge (green) and various cell trajectories in the experiment (left) and in cell simulations predicted by the three models (L to R, WP, WPI, and WPI-PIP3) with heterogenous parameters. In the lower part, all individual cell tracks are shown (25 for the experiment, 20 for each of the models), with the average trajectory overlaid and color-coded by rotation direction: blue for counterclockwise (CCW), red for clockwise (CW), and white for no rotation. In all models, the cell is first locally stimulated on its left from t = 100 s to t = 300 s, triggering counterclockwise rotation. At t = 300 s, global stimulus is turned on. WP predicts straight migration or loss of polarity and stalling; WPI continues its initial rotation; only WPI-PIP3 reverses direction, matching experimental results. Video shown in supplemental material: Video S4.

We found that if PIP3 dynamics are too fast, the peak dissipates before the inhibitor catches up, failing to account for the reversed direction of rotation. On the contrary, if PIP3 dynamics are too slow, Rac remains trapped near the PIP3 peak, yielding an oscillatory behavior. When we sample parameter sets from our fitted distributions (mimicking biological heterogeneity), we observe cases where reversal rotation does not occur, in agreement with experimental results. We briefly summarize these observations in Figures 14 and 15 and show details in supplemental information Figures S2 and S3.

Video S4. Comparasion of experiments with the WP, WPI, WPI-PIP3 models in the local-to-global stimulation pattern Only the WPI-PIP3 model is able to reproduce the experiment results.

### Interpretation of parameters obtained by fitting the time-dependent models

We now consider some implications of the parameters previously found by fitting the models to data.

(1) The basal Rac activation rate (*k*_*0*_) is small and much smaller than the elevated rate when Rac is autoactivating itself (*k*_*0*_ ≪ *γ*). Indeed, the basal rate is around 3% of the elevated activation rate (*k*_*0*_ ∼0.027 *γ*). This is required to assure a low resting-level of Rac activity that can get significantly elevated by positive feedback.
(2) The basal rate of Rac inactivation (*δ*) is considerably higher (by 3.75 fold) in the WP model that has no inhibitor. This makes sense, since in the WPI model, much of the work of damping out Rac activity is accomplished by the putative inhibitor.
(3) The level of total Rac (*T*) is larger in the WP model than in the WPI (34%). This difference can be understood by realizing that for long periods of stimulation, WP holds a steady level of activation, while WPI rises and then falls (following adaptation). Hence when fitting the models to data, WP was forced to balance between the peak and the long-term Rac activity, resulting in fits that favor higher values of the Rac activation rates. On the other hand, WPI can reach high peaks immediately after stimulation, followed by lower long-term activity, thanks to the slow inhibitor time dynamics.
(4) In the WPI model, the rate of decay of the inhibitor (*δ*_*H*_) is significantly lower (<1%) than the rate of decay of Rac. This implies that the inhibitor persists for much longer (half-life = ln(2)/0.0040–173 s), compared to Rac (half-life = ln(2)/0.721 ∼1 s).
(5) This is the first time that experimental data are being used to fit parameters to the WP model (since the Mori et al., 2008), and results of the fits are highly consistent with ball-park estimates made in that purely theoretical model. In particular, it was noted there and in Hughes et al.^17^ that a nonzero basal rate of Rac activation (*k*_*0*_ > 0) is theoretically essential for polarity by the WP mechanism to exist, but that it should be very small compared to the autocatalytic rate of Rac activation due to the positive feedback of Rac (*k*_*0*_ ≪ *γ*). The fits to the WP model, confirm this theory, even while the WPI model is seen to be a better fit to the neutrophil stimuli experiments.

It would be interesting to compare the parameter values with others from the literature, but the number of previous model fits to data is quite limited. Lockley et al.^18^^,^^19^ fit three polarity models^4^^,^^5^^,^^8^ to F-actin data from *Dictyostelium* cells repolarizing in response to reversal of a shear flow. These models do not specifically identify molecular components, but both Meinhardt’s^4^ and Otsuji’s^5^ models have an “active” variable (A, U, respectively). Otsuji^5^ has the pair U, V for active and inactive forms of the protein, with mass conservation, as does the Mori et al.^2^ WP model, though other details differ significantly. The leading rate of activation and decay of U in Otsuji et al. (*a*_*1*_∼0.38/s) and the inactivation rate of A in Meinhardt (*r*_*a*_ ∼0.23/s) are on the same order of magnitude as the rate *γ* for WP-Rac activation and the basal WPI-Rac inactivation rate *δ*. Other studies that adopt variants of the WP model, e.g., the Wang et al.^20^ cancer cell study, also use a speculative set of parameters, or at best, fit one or two parameter values to the model.

### Model cell responses to dynamic and noisy gradients

Having extracted meaningful parameter values from all experimental data, we can now test the responses of the model cell to virtual environments beyond those already tested experimentally, such as noisy and dynamic chemical gradients. To do so, we assumed that each point along the cell edge transduces the local attractant chemical concentration into an elevated rate of local Rac activation (this replaces the on-off 0–1 optogenetic light stimulus term previously used). The gradient of chemical results in a gradient of Rac activation, determining the Rac zone, the cell front, and hence, the direction and rate of migration.

**Figure 16:**
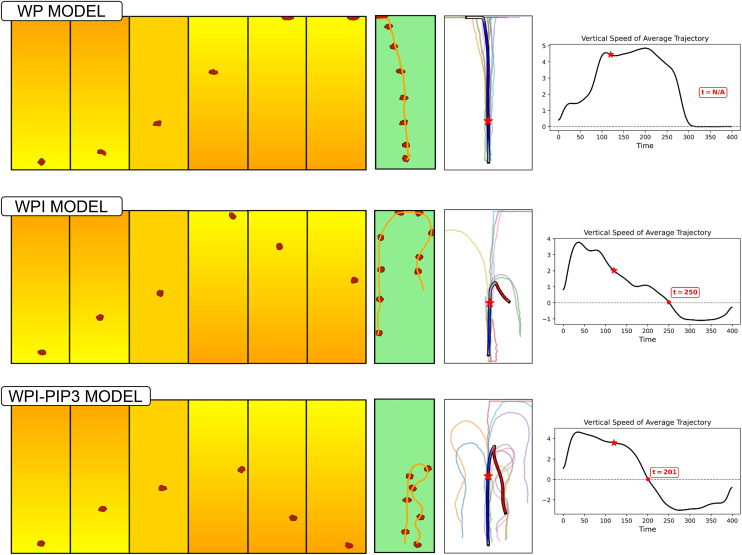
Model cell responses to dynamic chemotactic gradients Predicted sensitivity of the three types of model cells to dynamic chemical gradients. Left six images, snapshots of cell positions (red shapes) and chemical gradient from low (yellow) to high (orange) concentrations. In a noise-free gradient, eventually all cells align with the initial gradient. WP (top) polarizes very slowly and fails to reorient after gradient reversal. In contrast, WPI and WPI-PIP3 (middle and bottom) polarize faster and successfully reverse direction after the gradient flips. Green and white, single representative cell trajectory, followed by a sample of several. The blue-red colored track indicates the average path taken by the cells. The color changes from blue (northwards direction) to red (southwards direction), with a red star marking the gradient reversal timepoint. Right, average vertical velocity vs. time (with the same red star); the red dot indicates the cell reorientation time, (undefined for the WP model). Notably, WPI-PIP3 reorients faster (t = 201) than WPI (t = 250). The sudden drop in WP vertical speed is due to the cell reaching the top boundary of the domain, not active reorientation. Video shown in supplemental material: Video S5.

We simulated a scenario where cells are initially placed in a northward pointing chemical gradient. At *t* = 80, the chemical field is briefly reset to uniform, followed by gradient reversal at *t* = 120. As shown in Figure 16, all three models (WP, WPI, and WPI-PIP3) initially polarize and undergo chemotactic migration up the gradient. Based on our fitted parameters, we found that the WP model predicts delayed polarization onset compared with WPI and WPI-PIP3, particularly in regions of low chemoattractant concentration. Upon gradient reversal, only the inhibitor models (WPI and WPI-PIP3) successfully reoriented. WP remained fixed in its original direction. WPI cells showed a slower reversal, as shown by their short reversal paths. In contrast, WPI-PIP3 cells reversed more rapidly, producing longer reversed trajectories.

Video S5. Comparision between WP, WPI, and WPI-PIP3 models in the gradient stimulation instead of opto-stimulation This shows the flexibility that cells have in re-orient themselves when the gradient changes (only on WPI and WPI-PIP3).

The differences in the models can be explained as follows. First, the inhibitor accumulates at the front and destabilizes the Rac zone there. This destabilization facilitates the replacement of the existing front, making reversal more likely. Second, the faster reorientation in the WPI-PIP3 model arises from the PIP3 dynamic adaptation to the changing gradient. After the gradient reverses, PIP3 redistributes from a previously high level, decreasing across the cell, but more slowly in the direction of the new gradient. This creates an early asymmetry that facilitates a faster response.

Additional tests assessing model performance in noisy gradients are presented as rose plots in the supplemental information. These reinforce the same conclusion: the WPI-PIP3 model shows the most robust alignment across a variety of environmental conditions.

## Discussion

In this study, we developed and analyzed a series of basic, biologically grounded models for Rac activation and polarization in neutrophils. Our main contribution is to provide a modeling framework that validates a key experimental hypothesis proposed by Town and Weiner^1^ that a local Rac inhibitor is required to explain responses to optogenetic stimuli. Through a systematic investigation of several model variants, we demonstrated and explained the roles of a Rac inhibitor and of PIP3 in accounting for responses to various stimulation protocols, most importantly, the local to global stimulus experiment. These results show that combining a local inhibitory mechanism with a slow-adapting intermediate (PIP3) provides a robust and minimal mechanism for flexible polarity responses in motile cells.

The behaviors observed in our simulations suggest several biological implications. Most notably, we find that the ability to reorient in response to changing cues, such as a switch from local to global stimulation or a gradient reversal, requires both a local inhibitor and a dynamic “filtering” component such as PIP3. The inhibitor plays a primary role in enabling redirection by destabilizing the current front. PIP3 further enhances reorientation by smoothing stimulus transitions and preserving a directional bias after abrupt changes. This extended memory-like behavior helps the system avoid erratic responses, particularly in noisy environments. Together, the inhibitor and PIP3 work in concert, to provide a basic biologically plausible mechanism for robust polarity control in migrating cells.

Previous modeling studies have also investigated Rac dynamics in other amoeboid systems such as *Dicyostelium*.^21^^,^^22^ Several modeling studies have previously explored the dynamics of cell polarity and migration using reaction-diffusion systems coupled to deformable domains. A previous model closest to ours is Neilson et al.^7^ where a putative pseudopod activator and two inhibitors (local and global) were assumed in reaction-diffusion PDEs on a deforming closed curve representing the edge of a motile 2D cell. Their simulation method has the advantage of including the effects of edge curvature and “membrane tension” on the RD system. Our CPM is better at depicting fluctuating 2D cell shapes, while identifying specific model components and fitting to experimental data. Other works, such as Marée et al.,^23^ introduced a multiscale model of keratocyte motility that combines GTPase signaling with actin-driven protrusions. More recently, Liu et al.^24^ extended the WP framework to include source-sink mechanisms and actin feedback, simulating various patterning regimes and cell shape behaviors using a custom CPM implementation (our previous work on related models and simulations^17^^,^^24^^,^^25^^,^^26^ demonstrates that TWs and polar solutions persist in 2D as well as 1D.) In a parallel modeling framework, Camley et al.^27^ employed a phase-field approach to couple shape dynamics with cell signaling.

The rate of diffusion of the inhibitor was found to be smaller than that of active Rac. The diffusion ratio of 1.0:0.47 (active Rac: inhibitor) implies that the inhibitor could have MW roughly (1.0/0.47)^9^ = 9.6 times that of Rac. Since typical small GTPases are around 21 kDa, this implies the inhibitor MW is on the order of 200 kDa. This is in line with some of the larger GAPs, such as ARHGAP5 (172 kDa) and MYO9B (243 kDa). Alternately, the inhibitor could have protein-binding domains that lead to formation of complexes around that size, also a feature known in GAPs, or it could be sequestered by the cytoskeleton, which would reduce its mobility. Our parameter fitting results also imply that the inhibitor is recruited (or activated) at a very low rate by Rac, while also having a very slow rate of inactivation relative to Rac kinetics. These results may eventually lead to further identification of the likely molecular candidate(s) playing the role of the Rac inhibitor. This is one direction in which the model predictions suggest future experiments whose aim is to identify the Rac inhibitor.

We have made predictions for how the three regulatory motifs create distinct responses of model cells in chemical gradients. This goes beyond what has already been tested experimentally. These predictions are now being extended to more complex environments, where sharp turns or other decisions are required, and to further regulatory components such as Rho and its response to membrane tension.

While previous studies provide important insights into self-organization and shape change, our work differs in three key ways. First, we test specific hypotheses about the molecular circuit underlying polarity reversal, using both non-spatial and spatial simulations to account for experimental observations. Second, rather than relying solely on hand-tuned parameters, our model is quantitatively calibrated to experimental data, enabling us to extract parameter distributions across cells and thereby account for biological heterogeneity. Third, our use of the Morpheus platform makes the full CPM model openly accessible and easily reproducible, lowering the barrier for further exploration and validation.

### Limitations of the study

While our model reproduces key aspects of polarity reversal and directional adaptation, it simplifies several biological processes. In particular, the dynamics are simulated in a one-dimensional periodic domain, which removes a degree of freedom for movements of molecules (Morpheus does not currently support PDE solvers on deforming 2D or 3D domains, for which custom-built code is needed). We also do not include mechanical feedback from membrane tension which has been shown to act as a global inhibitor of Rac dynamics.^12^^,^^28^ Furthermore, the model assumes that polarity dictates movement, without feedback from cell deformation.^25^^,^^29^ The intracellular dynamics are also simplified, focusing on a minimal Rac-inhibitor-PIP3 circuit and omitting additional regulators such as Rho or Cdc42.^29^^,^^30^^,^^31^ These simplifications allow us to isolate and test for minimal requirements for polarity reversal but also point to natural directions for future model refinement.

## Resource availability

### Lead contact

Requests for further information or resources should be directed to and will be fulfilled by the lead contact, Jupiter Algorta (jupitera@math.ubc.ca).

### Materials availability

This study did not generate new materials.

### Data and code availability

All data and custom scripts are reported. All data and custom scripts are reported in the key resources table.

## Acknowledgments

This work was supported by the 10.13039/100000002National Institutes of Health grant GM118167 (O.D.W.), and by a 10.13039/501100000038Natural Sciences and Engineering Research Council of Canada (NSERC, Canada) discovery grant (L.E.-K.).

## Author contributions

J.A. performed all computational modeling, simulations, and data analysis. J.A. and L.E.-K. developed the research framework and met regularly to guide the project. J.A., L.E.-K., J.P.T., and O.D.W. jointly interpreted results and discussed model design and experimental alignment. J.A., L.E.-K., J.P.T., and O.D.W. wrote the manuscript, and all authors reviewed and edited it prior to submission.

## Declaration of interests

The authors declare no competing interests.

## Declaration of generative AI and AI-assisted technologies in the writing process

J.A. used ChatGPT to identify relevant coding packages and interpret some compilation errors. J.A. reviewed and edited the code as needed.

## STAR★Methods

### Key resources table

**Table:** 

REAGENT or RESOURCE	SOURCE	IDENTIFIER
**Software and algorithms**		

Custom Python scripts	This paper	See Zenodo DOI https://zenodo.org/records/15429635, https://zenodo.org/records/15429793
Morpheus (Cellular Potts Model framework)	Starruβ et al.^3^	https://morpheus.gitlab.io
Python	Python Software Foundation	https://www.python.org
Original neutrophil cell data from Town and Weiner^1^	Town and Weiner^1^	https://doi.org/10.1371/journal.pbio.3002307

### Method details

#### Mathematical modeling

We employed a system of partial differential equations (PDEs) to model the reaction-diffusion dynamics of Rac (active and inactive forms), a putative Rac-inhibitor, and Phosphatidylinositol (3,4,5)-trisphosphate (PIP3). The specific equations for the Wave-Pinning (WP), Wave-Pinning with Inhibitor (WPI), and WPI-PIP3 models are described in detail in the Supplemental Information.

#### Simulation framework

Simulations were performed using Morpheus, a modeling environment based on the Cellular Potts Model (CPM) using the software Morpheus.^3^ The reaction-diffusion systems were solved on a 1D periodic domain representing the cell edge. For multiscale simulations involving cell motility, the 1D reaction-diffusion system was coupled to the 2D CPM grid, where local concentrations of active Rac at the cell edge dictated the probability of membrane protrusion and retraction.

#### Modeling optogenetic stimuli

Optogenetic stimulation was modeled as a localized increase in the activation rate of Rac (for WP and WPI models) or the production rate of PIP3 (for the WPI-PIP3 model). The spatial profile and temporal duration of the stimulus terms (*S*) were defined to match the experimental protocols (local, global, and ramped stimuli) described in the Results.

### Quantification and statistical analysis

#### Parameter estimation

Parameter fitting was performed using the Differential Evolution algorithm to minimize the residual sum of squares (RSS) between model outputs and experimental time-series data. For temporal fitting, we utilized data from latrunculin-treated cells to fit reaction kinetic parameters (independent of diffusion). For spatial fitting, we utilized data from motile cells to estimate relative diffusion coefficients (Du,Dv,DH). To account for biological heterogeneity, parameters were fit to individual cell traces where possible, resulting in distributions of best-fit parameters (Table 1).

#### Model selection

To compare the performance of the WP and WPI models against the experimental data, we calculated the Akaike Information Criterion (AIC). The AIC score was computed using the residual sum of squares (RSS) and the number of free parameters for each model (*k*). A lower AIC indicates a preferred model that optimizes the trade-off between goodness-of-fit and model complexity. Specific AIC values and weights are reported in Table 3.

**Table:** Model selection

Model	AIC	ΔAIC	AIC weight	RSS	Samples
WPI	938.6	0	∼1.00	3,707.5	425
WP	1,160.3	221.7	∼0.00	6,335.8	425

Akaike information criterion (AIC) model comparison using the best-fit parameters for the temporal data corresponding to the 120 s gap. The AIC values were calculated using observations from all time-points (30, 60, 120, 240, and 480 s), highlighting that the WPI model provides a significantly better fit to the data than the WP model. See supplemental information for further details. Lower AIC, ΔAIC = 0, and an AIC weight near 1 indicate the preferred model.

#### Statistical reporting

Data are presented as mean ± standard deviation (SD) or as 95% confidence intervals where appropriate. The number of cells (n) used for fitting and analysis is indicated in the figure legends and Table 1 (e.g., 58 cell data for WP temporal fits, 81 cell data for WPI temporal fits).

## Footnotes

Supplemental information can be found online at https://doi.org/10.1016/j.isci.2026.114641.
